# Effect of QiShenYiQi Pill on Myocardial Collagen Metabolism in Rats with Partial Abdominal Aortic Coarctation

**DOI:** 10.1155/2015/415068

**Published:** 2015-03-11

**Authors:** Shichao Lv, Meifang Wu, Meng Li, Qiang Wang, Xiaojing Wang, Ling Xu, Junping Zhang

**Affiliations:** ^1^Department of Geriatric Medicine, First Teaching Hospital of Tianjin University of Traditional Chinese Medicine, 314 An Shan Xi Road, Nan Kai District, Tianjin 300193, China; ^2^Tianjin University of Traditional Chinese Medicine, 312 An Shan Xi Road, Nan Kai District, Tianjin 300193, China

## Abstract

This study investigated the effect of QiShenYiQi pill (QSYQ) on myocardial collagen metabolism in rats with partial abdominal aortic coarctation and explored its mechanism of action. A series of assays were used to detect the effect and mechanism of QSYQ on systolic blood pressure, heart mass index, left ventricle mass index, HYP, expression of PICP, PIIINT, and CTX-I in serum, MMP-1, and TIMP-1 expression in myocardium. We observed that QSYQ can reduce the rate of myocardial collagen synthesis and increase the rate of myocardial collagen degradation. It also effectively improved the degree of myocardial fibrosis in partial abdominal aortic rats and it had a tendency to have a greater effect with longer treatment duration, which is related to the mechanism of regulation of MMP-1 and TIMP-1 expression in the myocardial rat.

## 1. Introduction

Myocardial fibrosis is common in a variety of cardiovascular diseases, and it is also an important pathologic factor in a variety of cardiovascular events (including effects of heart failure, arrhythmia, and sudden cardiac death) [1]. Abnormal reconstruction of damaged heart tissue, characterized by myocardial fibrosis, is a core pathological change seen in many types of chronic cardiovascular disease [2]. Therefore, developing an effective drug treatment has become a focus of medical research into myocardial fibrosis. Currently, such research in western medicine has focused on the renin-angiotensin-aldosterone system (RAAS), especially on angiotensin-converting enzyme inhibitors (ACEIs), angiotensin II receptor blockers (ARB), and aldosterone antagonists, which are known to have a definite therapeutic effect [3]. However, traditional Chinese medicine (TCM) has the advantage of targeting many components of a system and providing more integrated regulation than modern medicine, which tends to target a single pathological link [4]. In targeting myocardial fibrosis, we try to treat cardiovascular disease early by TCM, so as to improve the cardiac microenvironment, promote steady recovery, and even inhibit or reverse the myocardial fibrosis.

QiShenYiQi is a TCM composed of* Radix Astragali*,* Radix Salviae miltiorrhizae*,* Radix Notoginseng*, and* Lignum Dalbergia Odorifera* [5, 6]. QiShenYiQi pill (QSYQ) is approved by China State Food and Drug Administration in 2003 for treatment of coronary heart disease, angina pectoris [7]. QSYQ enables a stable dosage form, of which the main effective ingredients are astragaloside, tanshinol, protocatechualdehyde, and ginsenosides Rg1 and Rb1 [8, 9]. Astragaloside is the main effective component of* Radix Astragali*, and its pharmacologic actions include regulating the immune system, protecting tissues and organs, and decreasing glucose [10]. It also has anti-inflammatory and antiviral properties. Tanshinol and protocatechualdehyde are the main effective components of* Radix Salviae miltiorrhizae*; their pharmacologic properties include myocardial and neural protection, inhibition of thrombosis, and enhancement of the immune function [11]. Ginsenosides Rg1 and Rb1 are the main effective components of* Radix Notoginseng*, and their pharmacological actions include dilating blood vessels, reducing myocardial oxygen consumption, inhibiting platelet coagulation agents, decreasing circulatory fat, scavenging free radicals, and acting as an antioxidant [12].

Hypertension is the main risk factor for heart disease and is associated with 50% of cases of cardiovascular disease. Myocardial fibrosis is the main pathological feature of high blood pressure, which causes heart damage, and is currently one of the most disabling diseases [13]. However, can QSYQ improve cardiac remodeling of rats with partial abdominal aortic coarctation? In the current study, we investigated the effect of QSYQ on myocardial collagen metabolism in rats with partial abdominal aortic coarctation and explored its mechanism of action.

## 2. Methods

### 2.1. Animals

Male Wistar rats weighing 180–200 g were purchased from the Laboratory Animal Center of the Academy of Military Medical Sciences (Beijing, China; certificate number SCXK(Jun)2012-0004). Rats were housed in groups (4/cage) with free access to a normal rat diet and clean drinking water. The study was carried out in strict accordance with the recommendations in the Guide for the Care and Use of Laboratory Animals of Institutional Animal Care and Use Committee of Tianjin University of Traditional Chinese Medicine. The protocol was approved by the Animal Ethics Committee of Tianjin University of Traditional Chinese Medicine (no. TCM-2011-015-E03), China.

### 2.2. Materials

The reagents used in the study were as follows: noninvasive blood pressure measurement device (Zhenghua Instrument Equipment Co., Ltd., Anhui, China, Type: ZH-HX-Z); signal acquisition system (Zhenghua Instrument Equipment Co., Ltd., Anhui, China, Type: MD3000); pentobarbital sodium (Baihao Biological Technology Co., Ltd., Tianjin, China); hydroxyproline kit (Jiancheng Biology Engineering Institute, Nanjing, China); ELISA kits for procollagen type I carboxyl terminal peptide (PICP), procollagen type III amino terminal peptide (PIIINT), and collagen C telopeptide type I (CTX-I) (Cusabio Biotech Co., Ltd., Wuhan, China); antimatrix metalloproteinase-1 (anti-MMP-1), antitissue inhibitor of metalloproteinases (anti-TIMP), streptavidin-biotin complex with peroxidase (SABC-POD) (rabbit IgG) ready-to-use kits, and DAB Chromogenic Reagent Kit (Boster Biological Engineering Co., Ltd., Wuhan, China); Ultrapure RNA Extraction Kit, HiFi-MMLV First Chain cDNA Synthesis Kit, UltraSYBR Mixture (with Rox), and DNase I (RNase-free) (Tasly Pharmaceutical Co., Ltd., Beijing, China); QSYQ pill (Tasly Pharmaceutical Co., Ltd., Tianjin, China; specification was 500 mg/bag; lot number Z20030139); and valsartan capsules (Novartis Pharma Ltd., Beijing, China; specification was 80 mg/tablets; lot number H20040217).

### 2.3. Rat Model with Partial Abdominal Aortic Coarctation

We established a rat model of pressure-overloaded myocardial fibrosis by creating partial abdominal aortic coarctation as described previously by Doering et al. [14]. Wistar rats were placed on an adaptive feeding routine for 3–5 days and fasted preoperatively for 12 h, with free access to drinking water. Each rat was weighed and then anesthetized with 3% pentobarbital sodium (45 mg/kg) by intraperitoneal injection. Once the rat was under anesthesia, as judged by disappearance of corneal reflection and decrease in muscle strength, it was placed on the operating table, and an area of approximately 3 × 5 cm in the center of the abdomen was shaved and disinfected with iodine. A longitudinal incision 2.0–2.5 cm in length, situated 0.5 cm under the left edge of the costal arch and 0.5 cm to the left of the abdominal midline, was made, and the layers separated to open the abdominal cavity. The intestines were moved to the right side of the abdominal cavity using gauze dipped in normal saline, while the stomach and spleen were pushed upwards to fully expose the abdominal aorta 0.5 cm above the right renal artery branches. The abdominal aorta was then freed, and a number 7 needle (0.7 mm in diameter) laid alongside of it. The abdominal aorta and needle were banded using number 4 surgical suture (0.3 mm in diameter); then the needle was subducted. The viscera were replaced, and 200,000 U of penicillin was dripped into the abdominal cavity. The abdominal muscle and skin were then sutured, and picric acid solution was smeared onto the wound in order to prevent the rat from biting the wound and causing infection. After surgery, the rats were kept warm and fed with a sugar and salt mixture (4 : 1 ratio) until they were wide awake. During the next 3 days, the rats were given penicillin (200,000 U/day) by intramuscular injection in case of infection. For the sham-operated control group, their abdominal aorta was not banded, but all other steps were the same.

### 2.4. Animal Groups and Treatment

At 4 weeks after the partial abdominal aortic coarctation surgery, the rats were randomized into four groups, with sixteen rats per group: (1) sham-operated control group (surgery without ligation, as described above, and administered isopycnic sterile distilled water by gavage), (2) model group (administered isopycnic sterile distilled water by gavage), (3) valsartan group (7.2 mg/kg by gavage), and (4) QSYQ group (135 mg/kg by gavage). Dosage of valsartan capsules and QSYQ was determined following the body surface area, in which equivalent dosage in rats is equivalent to 6.3 times of the clinical dosage in human [15].

Blood and tissue specimens were harvested at 4 (eight rats per group) and 8 (eight rats per group) weeks after treatment started. Each rat was weighed (body mass, BM) and then anesthetized with 3% pentobarbital sodium (45 mg/kg) by intraperitoneal injection. Blood was then taken from the abdominal aorta, then the chest was opened, and the heart irrigated with normal saline at 4°C. The blood sample was centrifuged at 3000 rpm for 10 min; then the serum was collected. The extracted myocardial tissue was blotted with filter paper; then we weighed heart mass (HM) and calculated heart mass index (HMI, HMI = HM/BM (mg/g)). We removed atrium and right ventricular, whereas retaining the left ventricle and interventricular septum, then weighed left ventricle mass (LVM), and calculated left ventricle mass index (LVMI, LVMI = LWH/BM (mg/g)). The part of the tissue was fixed in 4% neutral formaldehyde buffer solution and the remainder was cooled quickly in liquid nitrogen for cryopreservation.

### 2.5. Measurement of Arterial Systolic Blood Pressure

Systolic blood pressure was measured with noninvasive blood pressure measurement device (Zhenghua Instrument Equipment Co., Ltd., Anhui, China, Type: ZH-HX-Z). We preheated the device about 20 min, then set the heat chamber at 37°C, and adjusted the pressure calibrator. Each rat was weighed and put into the fixation device. We took the rat tail through the pressurized device and fixed it by nylon strap. Then, we gave a pressure charge to the pressurized device and used signal acquisition system (Zhenghua Instrument Equipment Co., Ltd., Anhui, China, Type: MD3000) to record the systolic blood pressure. This was repeated 3 times and we took the average of 3 measurements as the result.

### 2.6. Measurement of Hydroxyproline in Myocardium by Alkaline Hydrolysis

For measurement of myocardial hydroxyproline (HYP), myocardial tissue was removed from the liquid nitrogen, then 30–100 mg of myocardial tissue was weighed accurately, and alkaline hydrolysis was used to detect HYP. A blank tube (containing sterile distilled water) and a standard tube (containing 5 *μ*g/mL standard application solution) were also prepared using hydroxyproline kit (#A030-2, Jiancheng Biology Engineering Institute, Nanjing, China). The absorbance of each sample was analyzed at 550 nm by colorimetry, according to the manufacturer's instructions. The content of myocardial HYP was calculated using the following formula:

(1) HYPμg/mg=absorbancy  of  test  tube  −absorbancy  of  blank  tube ·absorbancy  of  standard  tube   −absorbancy  of  blank  tube−1 ×content  of  standard  tube5 μg/mL ×volume  of  hydrolysate(10 mL)wet  weight  of  tissue(mg).

### 2.7. Measurement of PICP, PIIINT, and CTX-I in Serum by ELISA

The level of serum PICP, PIIINT, and CTX-I was performed using ELISA kits (#CSB-E08081r, #CSB-E07927r, #CSB-E12776r, Cusabio Biotech Co., Ltd., Wuhan, China), according to the manufacturer's instructions.

### 2.8. Measurement of MMP-1 and TIMP-1 in Myocardium by Immunohistochemical Staining

Myocardial tissue was embedded in paraffin wax, cut into sections 5 *μ*m thick, and placed onto coverslips coated with poly-L-lysine. Coverslips were incubated for 1 hour at 60°C and then processed by conventional dewaxing. They were then placed in 0.3% H_2_O_2_ for 10 min to block endogenous peroxidase activity and washed three times with sterile distilled water. The sections were then placed into 0.02 M PBS (pH 7.2–7.6) and heated in a microwave to boiling point, after which the solution was left to cool naturally. 5% bovine serum albumin (BSA) (#AR0004, Boster Biological Engineering Co., Ltd., Wuhan, China) was added dropwise, and the slides were incubated for 30 min at 37°C later; then the excess liquid was shaken off without washing. The relevant primary antibody (rabbit anti-rat IgG) (anti-MMP-1: #BA1270-1, anti-TIMP-1: #BA3727, Boster Biological Engineering Co., Ltd., Wuhan, China) was added dropwise; then slides were incubated at 4°C overnight (at least 16 hours). Slides were washed with PBS (pH 7.2–7.6) three times, for 10 min each time. The secondary antibody (biotin-labelled goat anti-rabbit IgG) (#SA1022, Boster Biological Engineering Co., Ltd., Wuhan, China) was added dropwise and incubated at 30 min at 37°C and then washed with PBS (pH 7.2–7.6) three times, for 10 min each time. SABC reagent (#SA1022, Boster Biological Engineering Co., Ltd., Wuhan, China) was then added dropwise; slides were incubated for 30 min at 37°C and washed with PBS (pH 7.2–7.6) three times, 10 min each time. And then diaminobenzidine (DAB) staining (#AR1022, Boster Biological Engineering Co., Ltd., Wuhan, China) was performed following the manufacturer's instructions. The reaction was monitored under a microscope at room temperature, until being complete, and then washed with sterile distilled water. Slides were counterstained with hematoxylin, dehydrated in ethanol, cleared in xylene, mounted with neutral rubber sealant, and then observed under the microscope. Positive cells showed a clear structure and significantly darker staining than the background, with tan particles, whereas negative cells were either clear or not different from the background staining. Cells were counted in five microscopic fields chosen randomly at ×400 magnification under the microscope. Image analysis software (Image-Pro Plus 6.0) was used to calculate the ratio of positive area to whole area, and the average value was taken.

### 2.9. Measurement of MMP-1 and TIMP-1 in Myocardium by RT-qPCR

Total RNA was extracted from myocardial tissue (Ultrapure RNA extraction Kit (#CW0581, ComWin Biotech Co., Ltd., Beijing, China)). Absorbance was measured using an ultraviolet spectrophotometer and purification was evaluated by the ratio of absorbance at 260 and 280 nm (OD260/OD280). If the ratio ranges between 1.8 and 2.0, it indicates that the RNA is highly purified and suitable for real-time quantitative polymerase chain reaction (RT-qPCR).

Reverse transcription was carried out using a cDNA First Chain Synthesis Kit HiFi-MMLV (#CW0744, ComWin Biotech Co., Ltd., Beijing, China) and amplified using UltraSYBR Mixture (with Rox) (#CW0956, ComWin Biotech Co., Ltd., Beijing, China), according to the manufacturer's instructions. The forward MMP-1 primer was 5′-AATCCCATCCAGCCAACA-3′, and the reverse MMP-1 primer was 5′-ACAAACGGCAGCGTCAAT-3′ (354 bp product). The forward TIMP-1 primer was 5′-GCCTCTGGCATCCTCTTG-3′, and the reverse TIMP-1 primer was 5′-CTGCGGTTCTGGGACTTG-3′ (287 bp product). The housekeeping gene GAPDH (#RQP049537, Guangzhou Fulengen Co., Ltd., Guangzhou, China) was used as a control. Relative quantitative analysis of mRNA was carried out using the 2^−ΔΔCt^ method.

### 2.10. Statistical Analysis

All parameters were expressed as mean ± S.D. Statistical analysis was performed using one-way ANOVA followed by the least significant difference (LSD) test for multiple comparisons. SPSS statistical software (version 11.5, SPSS Inc., Chicago, IL, USA) was used for all statistical analyses. The level of significance was set at *P* < 0.05.

## 3. Results

### 3.1. Effect of QSYQ on Systolic Blood Pressure

**Figure 1:**
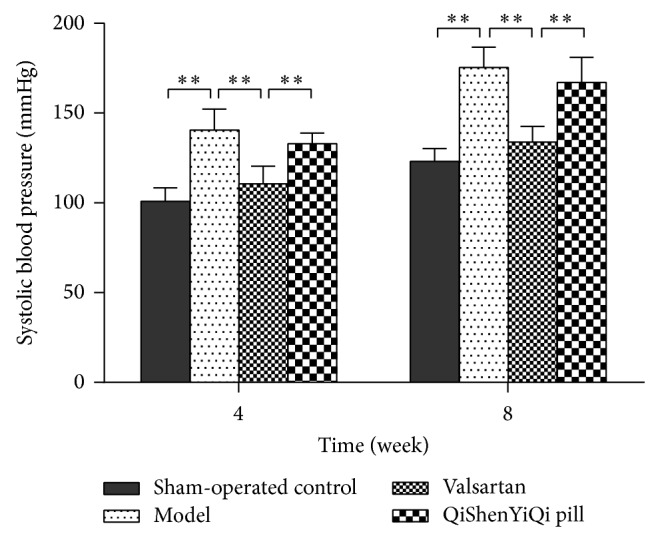
Effect of QSYQ on systolic blood pressure. Groups: control (*n* = 8), model (*n* = 8), valsartan (*n* = 8), and QSYQ (*n* = 8). Data are expressed as mean ± SD. ^**^ *P* < 0.01.

Compared with the sham-operated group, systolic blood pressure (SBP) was increased significantly in model group (*P* < 0.01) and showed a tendency to increase over time. SBP significantly reduced in the valsartan group compared with the model group (*P* < 0.01), while there was no statistical difference in the QSYQ group (*P* > 0.05). And SBP was lower in the valsartan group than in the QYSQ group (*P* < 0.01) (Figure 1).

### 3.2. Effect of QSYQ on HMI and LVMI

**Figure 2:**
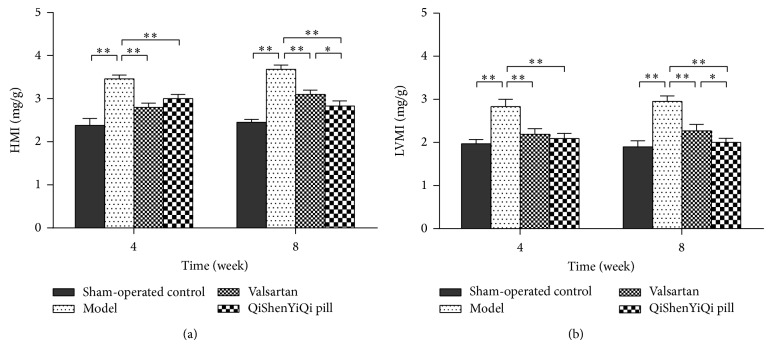
Effect of QSYQ on HMI and LVMI. (a) HMI of each group. (b) LVMI of each group. Groups: control (*n* = 8), model (*n* = 8), valsartan (*n* = 8), and QSYQ (*n* = 8). Data are expressed as mean ± SD. ^*^ *P* < 0.05, ^**^ *P* < 0.01.

Compared with the sham-operated group, the HMI and LVMI were increased significantly in model group (*P* < 0.01), and they increased further over time. With regard to the two treatment groups, the HMI and LVMI were significantly reduced in both the valsartan and the QSYQ group (*P* < 0.01), and this reduction was greater over time. But the HMI and LVMI were just lower in the QSYQ group than in the valsartan group at 8 weeks (*P* < 0.05) (Figure 2).

### 3.3. Effect of QSYQ on HYP Content

**Figure 3:**
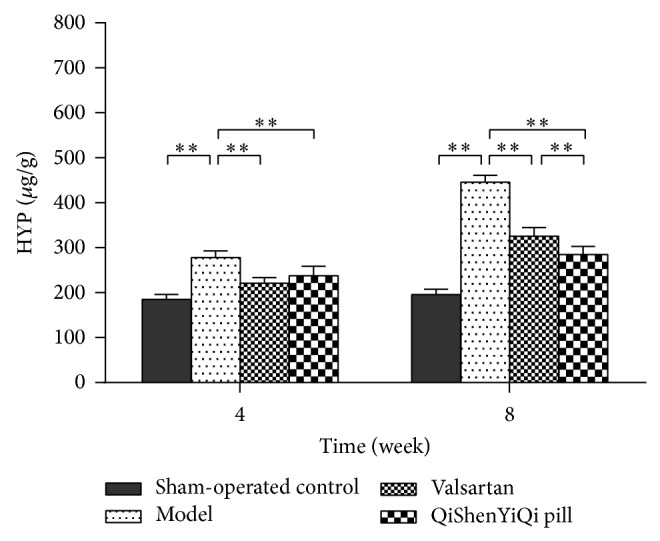
Effect of QSYQ on the content of HYP in rats with partial abdominal aortic coarctation. Groups: sham-operated control (*n* = 8), model (*n* = 8), valsartan (*n* = 8), and QSYQ (*n* = 8). Data are expressed as mean ± SD. ^**^ *P* < 0.01.

Compared with the sham-operated control group, the content of myocardial HYP was increased significantly in the model group at 4 weeks (*P* < 0.01), and it increased further over time, being higher again at 8 weeks (*P* < 0.01). With regard to the two treatment groups, the content of myocardial HYP was significantly reduced (*P* < 0.01) in both the valsartan and the QSYQ group after 4 weeks. Although the HYP content had risen by 8 weeks, it was lower in the QSYQ group than in the valsartan group (*P* < 0.01) (Figure 3).

### 3.4. Effect of QSYQ on the Synthesis and Degradation of Myocardial Collagen

**Figure 4:**
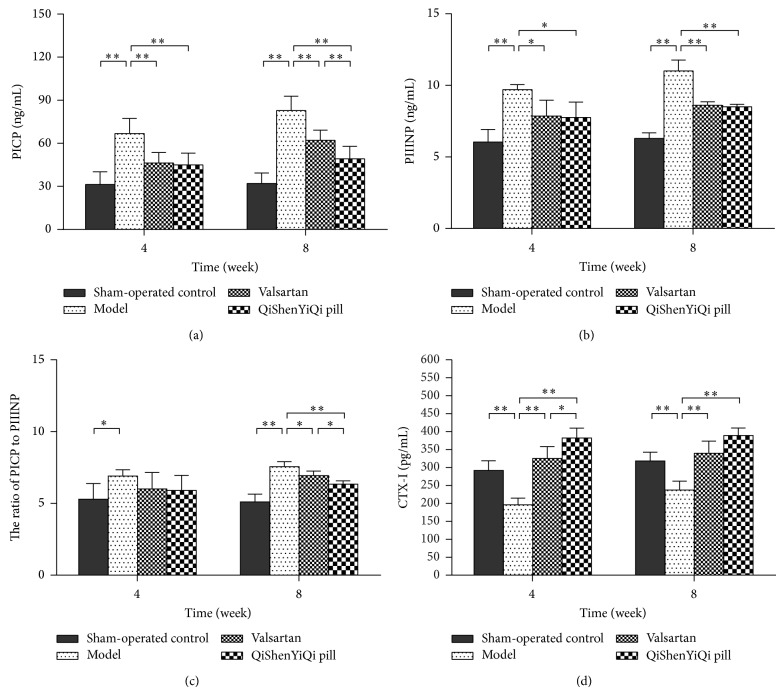
Effect of QSYQ on synthesis and degradation of myocardial collagen in rats with partial abdominal aortic coarctation. (a) Serum PICP levels of each group. (b) Serum PIIINP levels of each group. (c) The ratio of PICP to PIIINP. (d) Serum CTX-I levels of each group. PICP, PIIINP, and CTX-I were determined by enzyme-linked immunosorbent assay (ELISA). Groups: sham-operated control (*n* = 8), model (*n* = 8), valsartan (*n* = 8), and QSYQ (*n* = 8). Data are expressed as mean ± SD. ^*^ *P* < 0.05, ^**^ *P* < 0.01.

Compared with the sham-operated control group, the concentration of serum PICP and PIIINP and the ratio of PICP/PIIINP increased significantly (*P* < 0.01) in the model group and showed a tendency to increase over time. Valsartan significantly decreased the concentration of serum PICP and PIIIN (*P* < 0.01 and *P* < 0.05, resp.) and significantly decreased the ratio of PICP/PIIINP at 8 weeks (*P* < 0.05). A similar result was seen with the QSYQ group, except that its effect was significantly greater than that of valsartan at 8 weeks (*P* < 0.01, *P* < 0.05) (Figure 4).

Compared with the sham-operated control group, the concentration of serum CTX-I decreased significantly in the model group (*P* < 0.01). The concentration of serum CTX-I increased significantly (*P* < 0.01) in the valsartan group, and it showed a tendency to increase over time, while the QSYQ group showed a similar result, except that its effect was significantly greater than that of valsartan at 4 weeks (*P* < 0.01) (Figure 4).

### 3.5. Effect of QSYQ on Expression of MMP-1 and TIMP-1

**Figure 5:**
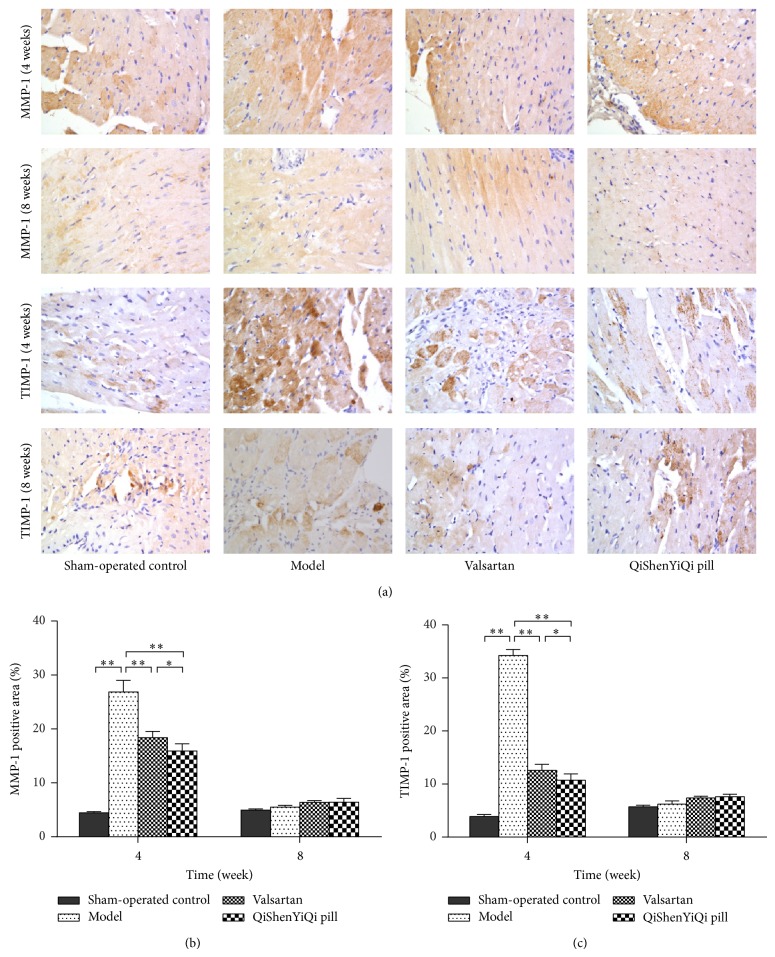
Effect of QSYQ on expression of MMP-1 and TIMP-1 in rats with partial abdominal aortic coarctation. (a) Representative photomicrograph of immunohistochemical staining of myocardium. (b) The MMP-1 positive area for each group. (c) The TIMP-1 positive area for each group. The percentage of immunohistochemical staining area was quantitatively analyzed by using Image-Pro Plus 6.0 (magnification, ×400). Data are expressed as mean ± SD. Groups: sham-operated control (*n* = 8), model (*n* = 8), valsartan (*n* = 8), and QSYQ (*n* = 8). ^*^ *P* < 0.05, ^**^ *P* < 0.01.

Immunohistochemical staining of MMP-1 and TIMP-1 in myocardial tissue after 4 weeks showed that, in the sham-operated control group, there were a small number of tiny tan particles scattered throughout the cytoplasm and the color change was weak, while, in model group, the brown area had expanded, staining was enhanced, and expression of MMP-1 and TIMP-1 had increased. After the treatments (valsartan and QYSQ), expression of MMP-1 and TIMP-1 was decreased compared with the model group at 4 weeks, whereas, at 8 weeks, there was no obvious difference in the brown areas between the control (sham-operated and model) and treatment (valsartan and QYSQ) groups (Figure 5).

Semiquantitative analysis showed that, after 4 weeks, the percentage positive area of MMP-1 and TIMP-1 protein was significantly increased (*P* < 0.01) in the model group compared with the sham-operated control group. The percentage positive area of MMP-1 and TIMP-1 significantly (*P* < 0.01) decreased in the valsartan group, while the QSYQ group had a similar result, except that the increase was significantly greater (*P* < 0.05) than that of the valsartan group at 4 weeks. At 8 weeks, there was no statistical difference between valsartan and QSYQ regarding the percentage positive area of MMP-1 and TIMP-1 (*P* > 0.05).

### 3.6. Effect of QSYQ on mRNA Expression of MMP-1 and TIMP-1

**Figure 6:**
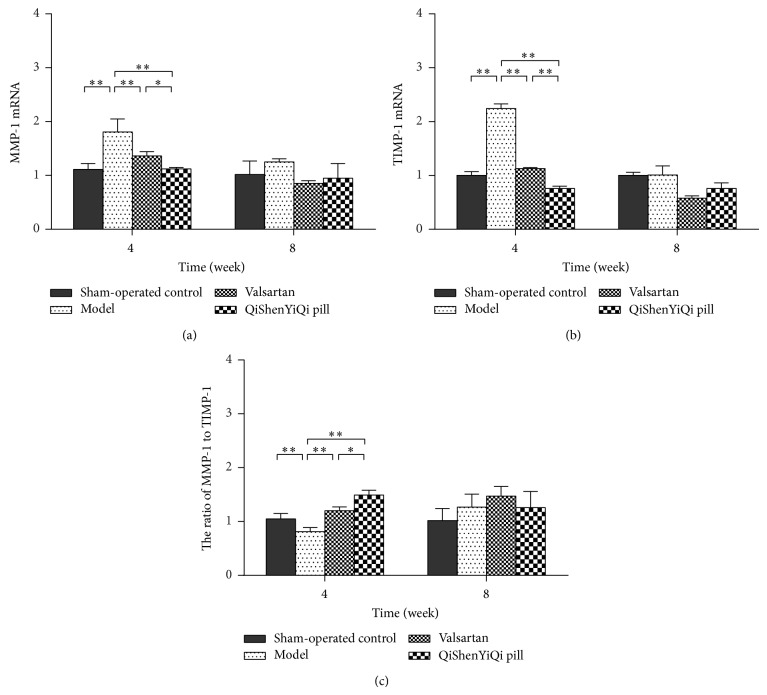
Effect of QSYQ on mRNA expression of MMP-1 and TIMP-1 in rats with partial abdominal aortic coarctation. (a) The MMP-1 mRNA expression for each group. (b) The TIMP-1 mRNA expression for each group. (c) The ratio of MMP-1 to TIMP-1. Groups: sham-operated control (*n* = 8), model (*n* = 8), valsartan (*n* = 8), and QSYQ (*n* = 8). Data are expressed as mean ± SD. ^*^ *P* < 0.05, ^**^ *P* < 0.01.

At 4 weeks, rat myocardial expression of MMP-1 and TIMP-1 mRNA was increased in the model group compared with the sham-operated control group (*P* < 0.01), but the ratio of MMP-1/TIMP-1 was decreased (*P* < 0.01). Valsartan produced a significant decrease in rat myocardial expression of MMP-1 and TIMP-1 mRNA (*P* < 0.01) and a significant increase in the ratio of MMP-1/TIMP-1 (*P* < 0.01). QSYQ produced a similar effect, but it was significantly greater than that of valsartan (*P* < 0.01) at 4 weeks. At 8 weeks, there was no statistical difference between valsartan and QSYQ regarding mRNA expression of MMP-1 and TIMP-1 and the ratio of MMP-1/TIMP-1 (*P* > 0.05) (Figure 6).

## 4. Discussion

Myocardial fibrosis is caused by various factors. It results in excess collagen fiber deposition, increase in the concentration of collagen and collagen volume fraction, significant changes in the collagen ratio, and disordered arrangement of collagen fibers [4]. HYP, which accounts for 13.4% of collagen, makes up a very small proportion of elastin and is almost nonexistent in the other proteins [16]. Thus, the content of myocardial HYP can be an indirect reflection of the level of myocardial collagen, and the content of myocardial HYP in fibrotic lesions is positively correlated with the degree of fibrosis [17]. Collagen content (collagen volume fraction) in normal rat myocardial tissue is about 3–5% in the rat; while the collagen content goes up to 8–12%, the diastolic stress-strain relationship is changed and diastolic function is damaged, but systolic function can be maintained [18]. However, when the content goes up to more than 20%, myocardial systolic function is weakened in the rat [18]. In the current study, after 4 weeks of treatment with QSYQ, the HMI, LVMI, and the content of myocardial HYP were significantly reduced in rats with partial abdominal aortic coarctation. Moreover, the degree of reduction was greater than that produced by valsartan at 8 weeks, indicating that QSYQ can improve myocardial fibrosis in rats with partial abdominal aortic coarctation and that this improvement becomes more significant with extended treatment time. But QSYQ had no effect on systolic blood pressure in rats with partial abdominal aortic coarctation, indicating that QSYQ improves myocardial fibrosis independently of blood pressure.

Cardiac extracellular matrix (ECM) contains the most abundant levels of collagen, with collagen type I and type III making up 85% and 11%, respectively, and the remainder being composed of collagen types IV and V [18]. The correct amounts and proportions of collagen types I and V are a precondition for maintaining normal structure and function of cardiac tissue. Imbalance between myocardial collagen synthesis and degradation causes a change in the proportion of collagens, with excessive deposition of collagen types I and III, resulting in myocardial fibrosis. Collagen type I mainly forms a thick fiber, and its stretch and rebound resilience are weak, while its stiffness is strong. By contrast, collagen type III mainly forms a slender fiber, and its stretch and rebound resilience are strong [19]. Increased levels of collagen type I can increase myocardial stiffness, while increased levels of collagen type III can increase ventricular compliance; therefore, the collagen type I/III ratio can be a good reflection of the degree of myocardial fibrosis [20]. In addition, a variety of myocardial collagen metabolism products, including PICP, PIIINP, and CTX-I, are involved in the process of myocardial fibrosis [21]. Clinical studies have shown that, compared with healthy controls, serum PICP levels are considerably higher in elderly patients with diastolic cardiac dysfunction, regardless of left ventricular hypertrophy. Serum PICP levels are significantly increased, which indicates that myocardial fibrosis is a major cause of diastolic cardiac insufficiency [22]. Myocardial collagen synthesis can be divided into two stages: procollagen synthesis in the cell and procollagen aggregation into collagen fibers outside the cell. Procollagen can form mutual cross-linking within the triple-helix structure of collagen fibers, while proteolytic enzymes cleave the amino and carboxyl terminal propeptides. PICP and PIIINP are precursors of myocardial collagens I and III polypeptides and free peptides, which are freed from procollagen during the process of procollagen being translated into collagen. PICP and PIIINP are indirect markers of myocardial collagen types I and III synthesis, and their levels increase in line with the increase in collagen synthesis [23, 24]. However, detecting the concentrations of PICP and PIIINP in the blood can only reflect the rate of collagen synthesis, not the rate of collagen degradation. CTX-I is the specific composition of collagen type I and is released into blood during the process of collagen type I degradation; it is therefore a marker of collagen type I degradation [25]. Research showed that serum CTX-I did not increase with the increase in collagen synthesis in spontaneously hypertensive rats [25], which indicates that collagen degradation may be insufficient and relative to a higher rate of collagen synthesis. Myocardial collagen metabolism markers are mainly used for assessing levels of myocardial fibrosis. Quantitative determination of the serum concentration of these markers can help to assess the degree of myocardial fibrosis, which is important in evaluating the effect of treatment and providing short-term and long-term prognoses. In our myocardial fibrosis model, established by pressure overload induced by abdominal aortic constriction, the serum levels of PICP and PIIINP were decreased and the serum level of CTX-I was increased after 4 and 8 weeks of treatment with both QSYQ and valsartan. However, QSYQ was better than valsartan at increasing CTX-I after 4 weeks and was also better than valsartan at decreasing PICP and PICP/PIIINP after 8 weeks, which indicates that QSYQ can reduce the rate of collagen synthesis and increase the rate of collagen degradation in myocardial fibrosis.

Excessive generation or abnormal degradation of cardiac ECM will damage the mechanical properties and structure of the myocardium, which will in turn affect the normal physiological functioning of the heart. Under normal physiological conditions, there is a balance between synthesis and degradation of the ECM, which is important to maintain the normal structure and function of tissues and organs. However, in pathological conditions, ECM synthesis increases and/or ECM degradation decreases, causing excessive accumulation of ECM and occurrence of fibrosis. MMPs are the most important enzymes for ECM degradation, while TIMPs are specific inhibitors of MMPs. The balance between MMPs and TIMPs maintains the stability of the cardiac collagen metabolism. MMP-1 is a collagenase and is mainly located in myocardial tissue. Its hydrolysis substrates are collagen types I, II, and III. The inhibitor of MMP-1 is TIMP-1, which is also located in myocardial tissue. TIMP-1 can affect the activity of MMP-1 by combining with it [26]. Mice with TIMP-1 genetic defects have obvious signs of ventricular remodeling and cardiac insufficiency after myocardial damage [27]. Clinical studies have shown that myocardial TIMP-1 and TIMP-2 mRNA expression is increased in patients with aortic stenosis [28], and myocardial MMP-1 and TIMP-1 protein expression is increased in patients with myocardial hypertrophy [29]. TIMP-1 expression was shown to be increased in a ventricular remodeling pig model induced by pressure overload [30]. Riociguat is a drug that alleviates left ventricular remodeling in salt-sensitive hypertensive rats by inhibiting TIMP-1, osteopontin, and plasminogen activator inhibitor-1 [31].

In patients with hypertension, there is a marked increase in serum TIMP-1 level [32]. Imbalance between MMPs and TIMPs will cause changes in cardiac structure and function and result in clinical manifestations in patients with hypertensions. An increase in TIMP-1 expression to >1200 ng/mL indicates congestive heart failure and is closely related to the degree of myocardial fibrosis [33, 34]. In the process of myocardial fibrosis induced by pressure load, a decrease in MMP-1 expression can prevent ventricular expansion, protect heart function, and increase the myocardial MMP 1/TIMP-1 ratio, which is an effective strategy to prevent hypertensive heart disease [35]. The correct proportion of MMPs and TIMPs is an important factor in maintaining normal heart structure [36]. When the MMP/TIMP ratio rises, ventricular remodeling develops towards ventricular expansion, whereas when the MMP/TIMP ratio decreases, the ventricular remodeling develops towards collagen accumulation [37]. In the current study, after 4 weeks of treatment with QSYQ, the mRNA and protein expression of MMP-1 and TIMP-1 decreased and the MMP/TIMP ratio rose. The effect of QSYQ was greater than that of valsartan. The study indicates that QSYQ can adjust the myocardial collagen metabolism in the abdominal aorta coarctation rat by regulating the expression of MMP-1 and TIMP-1.

## 5. Conclusion

QSYQ can reduce the rate of myocardial collagen synthesis and increase the rate of myocardial collagen degradation. It also effectively improved the degree of myocardial fibrosis in partial abdominal aortic rats and it had a tendency to have a greater effect with longer treatment duration, which is related to the mechanism of regulation of MMP-1 and TIMP-1 expression in the myocardial rat.
